# Management of right-sided obstructing colon cancers: scoping review

**DOI:** 10.1093/bjsopen/zraf129

**Published:** 2025-11-04

**Authors:** Daniel M Baker, Kelsey Aimar, Sam Jacobs, Matthew J Lee

**Affiliations:** Leeds Institute of Emergency General Surgery, St James's University Hospital, Leeds, UK; Department of Trauma and Emergency General Surgery, University Hospitals of Birmingham, Birmingham, UK; Department of Trauma and Emergency General Surgery, University Hospitals of Birmingham, Birmingham, UK; Department of Applied Health Sciences, College of Medicine and Health, University of Birmingham, Birmingham, UK

## Abstract

**Introduction:**

Patients presenting with right-sided obstructing colon cancers (ROCCs) typically undergo emergency surgical resection. Emerging evidence suggests using a bridge-to-surgery (BTS) approach with a stoma or stent reduces mortality and perioperative morbidity while not effecting long-term survival. The aim of this scoping review was to provide a comprehensive report of the recent literature reporting the management of ROCC.

**Methods:**

Systematic searches were conducted of the Embase, MEDLINE, and CENTRAL databases for studies published between 2000 and 2025. Searches were uploaded to the Covidence review tool and dual screened (abstract and full text) against prespecified inclusion criteria. Key data extraction points were: study characteristics, interventions used, patient and oncological descriptors, and outcomes reported. Data are reported descriptively as per PRISMA-ScR guidance.

**Results:**

Twenty-seven studies were identified: 22 primary research studies and 5 systematic reviews. Primary research studies were predominantly retrospective cohorts (20), with nine single-centre studies. Emergency resection was the most common intervention (7528, 75.4%), ahead of a colonic stent as a BTS (2289, 22.9%) and defunctioning stoma as a BTS (114, 1.1%). Of note, the vast majority of patients treated with a stent were from a single paper. There were 76 different descriptors reported, with 61 reported in fewer than five studies. In all, 70 perioperative, 8 stent-specific, and 21 oncological outcomes were reported. The focus was on perioperative outcomes, with only two oncological outcomes reported in five or more studies. Of the five included systematic reviews, four compared emergency resection to BTS and reported favourable outcomes of the BTS approach.

**Conclusion:**

Current available evidence is non-randomized and limited by retrospective study design. Although BTS appears favourable, studies are poorly powered, meaning the current evidence is insufficient to support BTS approaches.

## Teaser

Right-sided obstructing colon cancers are usually managed with emergency resection, which comes with high morbidity, limiting access to adjuvant chemotherapy. This study provides an up-to-date review of the current literature supporting alternative approaches via the bridge-to-surgery approach. These approaches appear favourable, but data are lacking to support these conclusions.

## Introduction

Colorectal cancer is the third most common cancer worldwide, with around two million new diagnoses per year^1^. Approximately 15–20% of patients are diagnosed acutely, most commonly due to bowel obstruction, with over 3000 emergency colon resections for colorectal cancer performed in the UK each year^2–4^. The management of obstructing colon tumours is complex, with one of the principal factors influencing surgical decision-making being anatomical location. Right-sided obstructing colon cancers (ROCCs) are traditionally treated with an emergency resection and anastomosis, with or without defunctioning ileostomy. The literature reports significantly higher mortality and morbidity rates, as well as reduced overall and disease-free survival, in emergency resections compared with elective resections^5–8^.

Patients presenting with ROCCs are often of advanced age, with more medical co-morbidities and a more locally advanced cancer than those undergoing elective resections^9^. This may explain, in part, the higher mortality rates; however, emergency resection has been found to be independently associated with decreased overall survival when adjusted for patient- and tumour-related factors^10^. This suggests a potential deficit in the perioperative management of emergency resections. There are statistically higher rates of T3 and T4 disease in ROCCs^9^, which should lead to recommendations of adjuvant chemotherapy to reduce the risk of cancer recurrence^11^. Despite this, access to chemotherapy following emergency resection has been found to be poor. Less than 30% of patients receive adjuvant treatment, with postoperative morbidity the primary reason cited^12^.

A bridge-to-surgery (BTS) approach can be used to avoid an emergency resection. This involves using a defunctioning stoma or a self-expanding metallic stent (SEMS) to relieve the initial obstruction before a definitive and expedited elective resection. This facilitates oncological staging, multidisciplinary team discussion, and medical optimization of the patient. Using a BTS approach has been investigated more thoroughly in left colon and rectal cancers, particularly the use of SEMS. The best available literature reports that SEMS as a BTS in left colon malignancies is oncologically safe, while decreasing mortality, improving rates of laparoscopic surgery, and decreasing the incidence of anastomotic leak and stoma formation^13–18^. Using a BTS approach in ROCCs also been found to have similar results, but the evidence is from lower-quality studies^19,20^.

Current data assessing BTS as an approach in ROCCs are lacking and often pre-date modern technological advances such as SEMS. In addition, the evidence for emergency resection often pre-dates the year 2000, and therefore limits interpretation given modern-day advances in surgical techniques, such as laparoscopic surgery in the emergency setting^21^.

The overall aim of this scoping review was to provide a comprehensive overview of the available literature reporting interventions in the management of ROCCs in modern surgical practice. This includes emergency resection and defunctioning stoma or SEMS as a BTS.

The questions this scoping review addressed are as follows:

- What are the characteristics of studies reporting outcomes of interventions following presentation with potentially curable ROCC?
- What descriptors are used to report patient, intervention, and oncological characteristics?
- What outcomes are reported for interventions used in ROCCs?

## Methods

### Protocol and registration

This scoping review was conducted in line with JBI evidence synthesis guidelines^22,23^ for scoping reviews and is reported using the PRISMA-ScR extension for scoping reviews framework^24^.

In line with these recommendations, scoping review protocols cannot be registered with PROSPERO. The protocol for this scoping review has been registered on the Open Science Framework *a priori*, as recommended by the JBI and is available online (https://osf.io/vk3fs/?view_only=d2521512a08f443cbe69bd10dc08d661).

### Search strategy

A comprehensive search strategy was created and performed by an information specialist via the Royal College of Surgeons of England support for systematic review searches. Searches were performed on January 2025 of MEDLINE (from 1946 to January 2025), Embase (from 1974 to January 2025), Cumulative Index to Nursing and Allied Health Literature (CINAHL), Cochrane Central Register of Controlled Trials (CENTRAL), and the Cochrane Database of Systematic Reviews (CDSR). The full search strategy is provided in *Tables S1–S3*.

### Eligibility criteria and definitions

The study inclusion criteria applied are listed below.

- Studies published in English.
- Studies including adult patients only (defined as aged ≥ 18 years).
- Studies of form, including retrospective/prospective cohort, randomized clinical trials, and systematic reviews with or without meta-analysis.
- Studies published after and inclusive of the year 2000. This was specified because it was the intent of the study to scope the most recent literature given SEMS was not used as an intervention until the 1990s. In addition, there have been advances in surgical techniques, such as the use of laparoscopic surgery in the emergency setting. The aim of this scoping review was to report the literature that reflects modern-day techniques, and the authors felt this was best achieved by restricting data to papers published in the 21st century. Where papers were published after 2000, but contained cohort data inclusive of a period before 2000 (for example, 1997–2003), a maximum of 5 years before 2000 was set as an arbitrary cut-off for exclusion. Systematic reviews were treated by year of publication.
- Studies reporting outcomes for obstructing cancers of the right colon, defined as proximal to the splenic flexure and distal to the ileocecal valve. If studies reported outcomes for obstructing colorectal tumours of differing anatomical locations, they were only included if the right-sided specific outcomes could be isolated and extracted.
- Studies reporting any post-procedural outcome for patients who were emergency presentations and were treated with curative intent. Curative intent was defined as all treatment that was not specified as a palliative approach within the study findings. This includes emergency resection with or without defunctioning ileostomy or defunctioning ileostomy/SEMS alone acting as a BTS.

### Article screening and selection

Articles that were identified by the initial searches were uploaded to Covidence systematic review software (Veritas Health Innovation, Melbourne, Victoria, Australia) and duplicates were removed. All abstracts were dual screened by two researchers (K.A., S.J.) against the specified inclusion criteria, with conflicts resolved by a third reviewer (D.M.B.). Full texts were retrieved and the screening process repeated. At this stage, included systematic reviews were hand searched for references and additional references were added to full-text screening and considered for final inclusion. The grey literature was not searched because this does not inform surgical practice.

### Data extraction

A data collection pro forma was created in Microsoft (Microsoft, Bellevue, WA, USA) Excel before data extraction. Data collection items represented the aims of this study and included baseline study characteristics (sample size, year of publication, country of origin, type of study), study descriptors used to report the study population (for example, patient demographics or histopathological descriptors), and postintervention and oncological outcomes reported by each study. Systematic reviews with or without meta-analyses were treated as a separate entity with the main aim and key points reported only. This is because each systematic review was hand searched for the original research papers to screen against the present study’s inclusion criteria.

The data extraction sheet was trialled by two authors (K.A., S.J.) on five papers before use and was refined accordingly. There was no planned assessment of study quality in this scoping review because there was no planned synthesis of the data, and assessment of bias is beyond the remit of a scoping review^22^.

### Data analysis

There was no planned meta-analysis of the results collected in this study. Data are reported narratively for key outcomes. Discussion was held between all authors as to how to name collated descriptors and outcomes to ensure outcomes of interest were grouped appropriately. Where outcomes were reported in five or more studies, quantitative data are reported as a median and a range (minimum-maximum) in support of reporting evidence related to the aims of this study.

## Results

### Search results

**Fig. 1:**
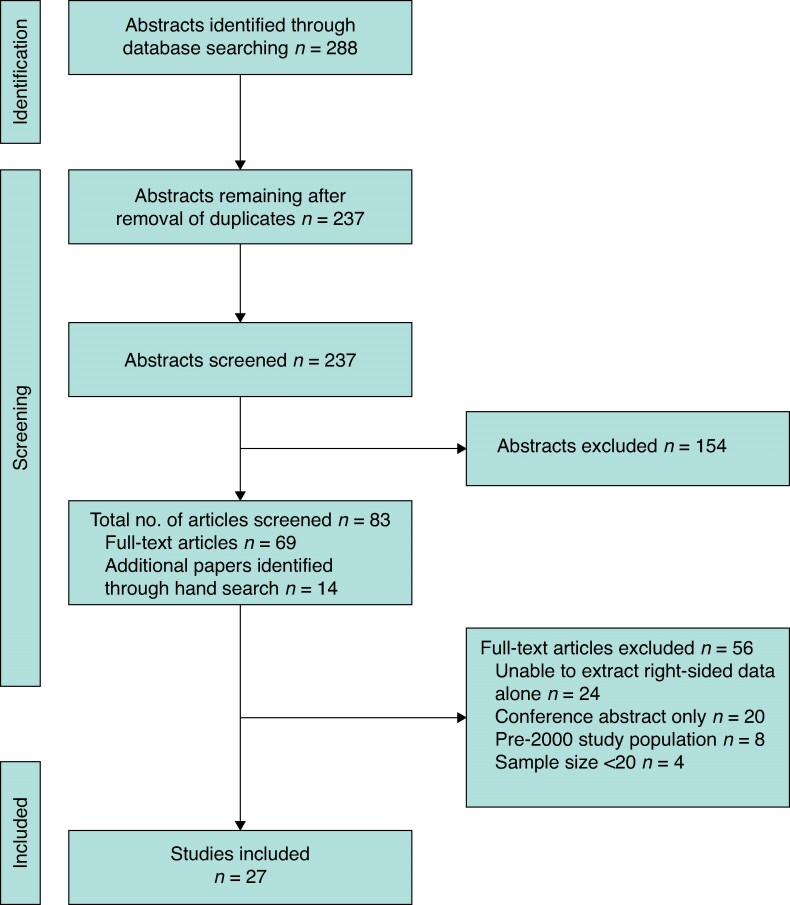
PRISMA flow chart

The study selection process is summarized in *Fig. 1*. In all, 288 papers were identified from initial searches, with 237 remaining after the removal of duplicates. After abstract screening, 69 papers met the inclusion criteria for full-text screening. This included five systematic reviews, which were hand searched and yielded an additional 14 papers. Thus, 83 papers were subjected to full-text screening, of which 27 were retained for data extraction (22 primary research papers, 5 systematic reviews). The systematic reviews were discussed separately.

Overall, 9979 patients were included from primary research studies, with 7528 (75.4%) undergoing emergency surgical resection, 2289 (22.9%) undergoing SEMS as a BTS, 114 (1.1%) undergoing defunctioning stoma as a BTS, and 48 (0.5%) undergoing defunctioning stoma of unclear treatment approach. The overwhelming majority of patients (1866) receiving SEMS as a BTS are from a single paper^25^, with the remaining 423 patients split over 12 studies, all of which had a SEMS sample size of <100 patients. Three studies reported outcomes of defunctioning stoma as a BTS, with sample sizes of 70^26^, 42^27^, and 2^28^, respectively.

Most papers were retrospective cohort studies (20), and of these 11 studies used multicentre data. Studies were from Asia (13) and Europe (9). Notably there were no US- or UK-based studies within the final data set. A summary of the characteristics of the primary research study is provided in *Table 1*.

**Table 1:** Characteristics of included primary research studies

Authors	Country of origin	Study type (study period)	Single centre or multicentre	Sample size (*n*)	Sample size by intervention (*n*)	Right colon cohort only?	Primary comparator (if present)
Amelung *et al*.^27^ (2016)	Netherlands	Prospective cohort (2009–2013)	Multicentre	1860	Emergency resection: 1774 Stoma as BTS: 42 SEMS as BTS: 44	Yes	Emergency resection *versus* BTS
Boeding *et al*.^29^ (2023)	Netherlands	Retrospective cohort (2013–2019)	Single centre	54	Emergency resection: 53 SEMS as BTS: 1	Yes	Optimized *versus* non-optimized patient (short-term outcomes)
Faucheron *et al*.^30^ (2018)	France	Prospective cohort (2000–2009)	Single centre	31	Emergency resection: 31	No	Left- *versus* right-sided emergency resections
Genser *et al*.^31^ (2020)	France	Retrospective cohort (2000–2015)	Multicentre	445	Emergency resection: 445	No	Operative outcomes in patients with obesity *versus* overweight *versus* normal weight
Hotta *et al*.^28^ (2012)	Japan	Retrospective cohort (1997–2008)	Single centre	38	Emergency resection: 36 Stoma as BTS: 2	No	Bowel decompression *versus* no decompression before emergency resection
Huang *et al*.^32^ (2024)	China	Retrospective cohort (2010–2021)	Single centre	95	Emergency resection: 60 SEMS as BTS: 35	Yes	Emergency resection *versus* BTS
Ji *et al*.^33^ (2017)	Korea	Retrospective cohort (2006–2014)	Multicentre	39	Emergency resection: 25 SEMS as BTS: 14	Yes	Emergency resection *versus* BTS
Kye *et al*.^34^ (2016)	Korea	Retrospective cohort (2004–2010)	Multicentre	74	Emergency resection: 49 SEMS as BTS: 25	Yes	Emergency resection *versus* BTS
Li *et al*.^35^ (2020)	China	Retrospective cohort (2008–2016)	Single centre	107	Emergency resection: 72 SEMS as BTS: 35	Yes	Emergency resection *versus* BTS
Li *et al*.^36^ (2015)	China	Retrospective cohort (2011–2013)	Single centre	35	Emergency resection: 35	Yes	Laparoscopic *versus* open emergency resection
Lockhorst *et al*.^37^ (2025)	Netherlands	Retrospective cohort (2013–2020)	Single centre	62	Emergency resection: 62	Yes	Optimized *versus* non-optimized patient (long-term outcomes)
Mege *et al*.^38^ (2018)	France	Retrospective cohort (2000–2015)	Multicentre	776	Emergency resection: 776	Yes	
Mege *et al*.^39^ (2019)	France	Retrospective cohort (2000–2015)	Multicentre	791	Emergency resection: 716 Defunctioning stoma: 48 SEMS as BTS: 27	No	Left- *versus* right-sided emergency resections
Morita *et al*.^40^ (2019)	Japan	Retrospective cohort (2014–2016)	Multicentre	68	Emergency resection: 40 SEMS as BTS: 28	No	SEMS as BTS in left *versus* right colon cancer
Ng *et al*.^41^ (2008)	Japan	Retrospective cohort (2003–2006)	Single centre	43	Emergency resection: 43	Yes	Laparoscopic *versus* open emergency resection
Rosander *et al*.^26^ (2021)	Sweden	Retrospective cohort (2007–2017)	Multicentre	751	Emergency resection: 681 Stoma as BTS: 70	No	Emergency resection *versus* BTS
Sakamoto *et al*.^25^ (2020)	Japan	Retrospective cohort (2012–2017)	Multicentre	4235	Emergency resection: 2369 SEMS as BTS: 1866	Yes	Emergency resection *versus* BTS
Suzuki *et al*.^42^ (2013)	Japan	Retrospective cohort (2007–2017)	Single centre	40	Decompression tube as BTS: 21 SEMS as BTS: 19	Yes	Decompression tubes as BTS *versus* SEMS as BTS
Tan *et al*.^43^ (2010)	Singapore	Retrospective cohort (2003–2008)	Single centre	27	Emergency resection: 27	No	Left- *versus* right-sided emergency resections
van den Berg *et al*.^44^ (2014)	Netherlands	Retrospective cohort (2005–2012)	Multicentre	33	Emergency resection: 17 SEMS as BTS: 16	No	Emergency resection *versus* BTS
Yao *et al*.^45^ (2011)	China	Retrospective cohort (2004–2010)	Single centre	81	SEMS as BTS: 81	Yes	
Zeng *et al*.^46^ (2021)	China	Retrospective case-control (2005–2015)	Multicentre	294	Emergency resection: 196 SEMS as BTS: 98	Yes	Emergency resection *versus* BTS

BTS, bridge to surgery; SEMS, self-expanding metallic stent.

Eight studies contained mixed-cohort data, including patients with both right- and left-sided obstructing colon cancers. Twenty studies had a primary aim that involved comparison between different treatment arms. Most commonly, this was emergency resection *versus* BTS with either stent or stoma (9 studies). Other comparisons used by more than one study included outcomes of left- *versus* right-sided emergency resection (3 studies), right-sided emergency resections in optimized *versus* non-optimized patients (2 studies), and laparoscopic *versus* open emergency resections (2 studies).

### Study descriptors

There was interstudy variability in the number of descriptors reported within a study, with a median of 7 (range 3–16). In all, 76 distinct descriptors were identified that were grouped into three categories: patient admission descriptors (26), intervention specific descriptors (27), and surgical oncology and histopathological descriptors (23). Only 16 descriptors were used in five or more studies and these were spread across patient descriptors (5), intervention descriptors (5), and histopathological descriptors (6), as shown in *Table 2*. The patency of the ileocecal valve was not used as a descriptor in any of the included papers. A full list of the descriptors is provided in *Table S4*.

**Table 2:** Summary of study descriptors reported in five or more studies

	No. of reporting studies	References
**Patient descriptors**		
Age	21	27–30, 32–46
Sex	20	27–30, 32–46
ASA score	14	26, 27, 29, 30, 32–34, 37–41, 43, 46
Co-morbidity	9	25, 32, 35, 37–39, 41–43
BMI	9	25, 32, 33, 36–38, 41, 42, 46
**Intervention/technicality descriptors**		
Surgical approach (open/laparoscopic/conversion to laparotomy)	14	25, 27, 29, 32–37, 40–42, 44, 46
Surgical procedure performed	10	27, 29, 31, 32, 37, 40, 41, 43–45
Duration of operation	9	32–36, 40–42, 46
Blood loss	9	32–36, 40–42, 46
Stoma formation	9	25–27, 32, 33, 35, 40, 43, 46
**Surgical oncology and histopathological descriptors**		
Staging	14	25, 28, 30, 32, 33, 35, 36, 38, 40–44, 46
No. of harvested lymph nodes	12	26, 30, 32–36, 38, 41, 42, 44, 46
Tumour location	11	25, 27, 29, 32, 33, 35, 40, 42, 43, 45, 46
Lymphatic invasion	6	28, 30, 34, 35, 38, 42
Vascular invasion	6	28, 30, 32, 34, 35, 42
Resection (R0/R1/R2)	5	27, 30, 35, 40, 44

ASA, American Society of Anesthesiologists; BMI, body mass index.

### Outcomes, excluding oncological outcomes

**Fig. 2:**
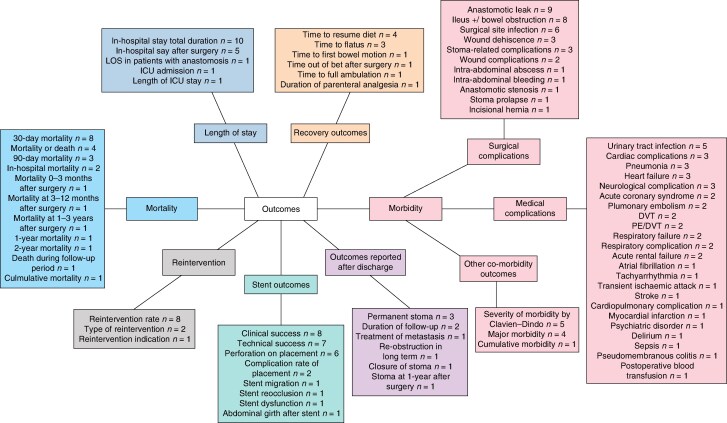
Spider diagram of reported outcomes Numbers in parentheses indicate the number of studies reported the outcome. ICU, intensive care unit; LOS, length of hospital stay; PE, pulmonary embolism; DVT, deep vein thrombosis.

There were 78 different outcomes reported. These were subdivided into mortality (11), morbidity (39), reintervention (3), length of hospital stay (5), patient recovery (6), stent-specific outcomes (8), and outcomes reported after discharge (6). Outcomes were reported with varying frequency, with 14 reported in ≥ 5 studies. There was marked interstudy variability, with a median of 14 outcomes per study, ranging from 3^28^ to 34^46^. The variety of non-oncological outcomes reported is shown in *Fig. 2*.

There were observed differences in how outcomes were reported and defined, in particular mortality, where there were 11 variations reported. Thirty-day mortality was most commonly reported (8), followed by ‘mortality’ with no specified time frame, although it was assumed to be short term (4). There was a varying incidence in the reported short-term mortality rates, with a tendency towards higher mortality within the emergency resection cohort than the BTS cohort (median 6.3% (range 0–18.5%) *versus* 2.5% (range 0–6.3%), respectively). Rates of anastomotic leak were reported as low as 0% for both emergency resection and BTS, with the highest incidence of anastomotic leak reported as 6.3% for BTS^44^ and 14% for emergency resection^38^.

### Oncological outcomes

**Fig. 3:**
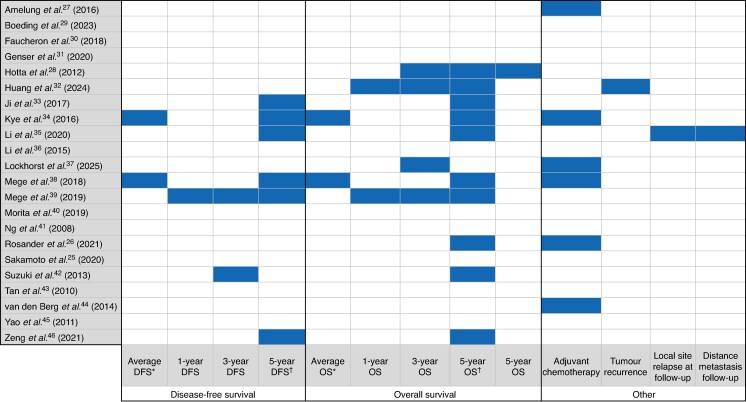
Oncological outcomes heat map *Includes studies reporting two versions of average (median and mean). †Includes studies reporting three separate versions of 5-year OS (OS, OS stage II, and OS stage III). DFS, disease-free survival; OS, overall survival.

There were 19 oncological outcomes reported across 13 studies, 13 of which were reported only once (*Fig. 3*). Five-year overall survival was reported in 10 studies; however, the reported rates were subject to significant variation. The reported 5-year overall survival rate within the emergency resection group ranged from 25%^26^ to 76.7%^32^. The BTS 5-year overall survival rates were also variable, ranging from 32%^42^ to 90.7%^34^. Only one of these papers^26^ had a sample size of more than 100 patients. The rate of adjuvant chemotherapy was reported by six papers ^26,27,34,37,38,44^, with rates of 29–67% after emergency resection, 27.9–68% for SEMS as BTS, and 38.1–55.7% for stoma as BTS. One study reported a higher observed rate of adjuvant chemotherapy in patients who were optimized before emergency surgical resection as opposed to immediate surgical intervention^37^.

### Timing to elective resection after the BTS approach

Three studies^34,35,45^ that reported SEMS as a BTS did not report the timing of elective surgery following stent placement, but did specify that the usual practice is within 7–14 days. The remaining studies reporting SEMS demonstrated expedited elective surgery, with median intervals to elective resection ranging from 6 to 28 days. There were outlying patients within studies, with a lowest reported wait of 2.5 days^44^ and a maximum of 118 days^27^. The timing of elective surgery after defunctioning stoma as a BTS was reported in two studies^26,27^ and demonstrated a longer wait before definitive resection compared with SEMS. One study^26^ reported a median of 69 (range 19–231) days, whereas the other^27^reported a mean average of 109.0 (range 0–358) days. It is not explained why there is a lower limit of 0 days, but it is assumed that the surgical approach changed during surgery.

### Systematic reviews

There were five systematic reviews identified^19,20,47–49^, four of which compared emergency resection to the BTS approach^19,20,47,48^, and three that conducted a formal meta-analysis^20,48,49^. A summary of these studies and key findings are presented in *Table 3*. All four studies reporting BTS outcomes describe a statistically lower mortality rate associated with BTS and elective resection *versus* emergency resection. Three studies^19,47,48^ report lower morbidity, specifically lower rates of anastomotic leak and stoma formation, in BTS. However, one study^20^ reported similar rates between groups, although higher rates of laparoscopic surgery were observed in the BTS group in that study. Others also reported stent specific outcomes. The stent success rate was 92% with low observed heterogeneity and the stent perforation rate was 3% with high levels of heterogeneity ^20^.

**Table 3:** Summary of included systematic reviews

Authors	Sample size of interventions (*n*)	Primary aim	Summary of key findings
Amelung *et al*. (2015)^19^	Emergency resection: 2837 SEMS as BTS: 155	Procedure-related mortality and morbidity rates of emergency resection *versus* SEMS as BTS before elective resection	Statistically lower mortality in the BTS group Statistically lower major morbidity in the BTS group
Boeding *et al*. (2021)^47^	Emergency resection: 515 SEMS as BTS: 77 Ileus tube decompression: 8	Systematic review of outcomes of patients presenting with obstructing right colon cancer with curative intent in post-1990 literature	BTS approach had lower mortality and few complications, including anastomotic leaks Five-year disease-free and overall survival rates were comparable between groups
Cirocchi *et al.* (2017)^49^	Emergency resection: 78	Comparison and meta-analysis of outcomes of open *versus* laparoscopic approach to emergency resections	Small sample included within meta-analysis (78 patients) Significantly lower 30-day complication rate in laparoscopic group Significantly lower LOS, intraoperative blood loss, and time to mobilization in laparoscopic group
Kanaka *et al.* (2022)^48^	Emergency resection: 3474 BTS (approach not specified): 1662	Evaluation of safety and effectiveness with a meta-analysis of emergency resection *versus* BTS, specifically assessing short-term outcomes	Significant reduction in mortality and postoperative complications with BTS *versus* emergency resection
Mader *et al.* (2023)^20^	Acute resection: 4201 SEMS as BTS: 2142	SEMS as a BTS *versus* emergency resection with a focus on a meta-analysis of outcomes; stent success rate, morbidity, mortality, stoma formation, laparoscopic resection and anastomotic leak	SEMS as a BTS led to statistically higher rate of laparoscopic surgery Anastomotic leak and primary anastomosis rates similar between groups Statistically higher mortality rate with emergency resection *versus* SEMS Stent success rate of 92% with low heterogeneity Complication rate of 5% and perforation rate of 3% with high heterogeneity

SEMS, self-expanding metallic stent; BTS, bridge to surgery; LOS, length of hospital stay.

## Discussion

This study provides a scoping review of the most up-to-date literature reporting outcomes of interventions used in the management of patients with potentially curable ROCCs. The results of this study highlight the lack of high-quality data available to provide an understanding of outcomes in modern-day surgical practice. To compound this, the studies that do exist report findings inconsistently, with a wide range of descriptors and outcomes used within studies. A BTS approach appears to demonstrate favourable postoperative outcomes, but this interpretation is significantly guarded given the small sample sizes and lack of high-quality data from randomized trials.

Traditionally, the management of ROCCs has been immediate surgical resection, which comes with high mortality and morbidity^7^. Definitive surgical resection forms only part of the treatment for ROCCs, with oncological treatment in the form of adjuvant chemotherapy also crucial to definitive management^11^. Access to adjuvant chemotherapy following emergency resection is known to be poor due to the associated morbidity of major emergency surgery. This subsequently affects oncological outcomes in these patients^12^, highlighting the need for the consideration of an alternative approach in the emergency setting to improve patient outcomes. Recent oncological data from the FOXTROT trial have demonstrated that a short course of neoadjuvant chemotherapy in T3–T4 colon cancers can lead to histopathological downstaging and improved complete (R0) resection rates and 2-year disease-free survival^50^. ROCCs are often locally advanced (T3/T4) at presentation and, in view of the FOXTROT trial findings, it is plausible to suggest that these patients may also benefit from neoadjuvant chemotherapy if the emergency obstruction can be relieved without significant morbidity. It is therefore essential that high-powered research is conducted to truly assess the feasibility of BTS approaches in practice, including oncological outcomes.

The use of SEMS in the management of obstructing colon cancers was first established in the palliative setting in the early 1990s^51^, but its use in the proximal colon did not arise until 1997^52^. The use of SEMS has been more rigorously researched in left colon cancers owing to the anatomical location being more easily accessible endoscopically. SEMS as a BTS in obstructing left colon cancers has been found to reduce mortality, morbidity, and stoma rates while demonstrating equivocal long-term oncological outcomes^13,14,16,18,53^. International guidelines are variable in recommending SEMS^54^, but recent UK^11,55^ and US guidelines^56^ recommend SEMS as an equivocal treatment to emergency surgery and suggest a shared decision-making approach.

Similar results have also been demonstrated in right colon cancers, with authors supporting the use of SEMS in proximal cancers^57–59^. Despite this, the World Society of Emergency Surgery^60^, UK^11,55^, and European^61^ guidelines do not support SEMS in ROCCs, although US guidance does^56^. The results of the present study highlight the difficulties in interpreting the literature and give context to these recommendations. Within this review, there were a number of studies reporting outcomes of SEMS, but only one of these reported on a sample size greater than 100 patients^25^. The variability in descriptor and outcome reporting identified in this study also creates a major barrier to interpretation and, although there have been attempts to perform a meta-analysis on the literature, heterogeneity is high because of these variations.

However, the present study does demonstrate the potential utility of a BTS approach. The 5-year overall survival in this study was variable, but BTS had better observed rates at both the upper and lower range boundaries compared with emergency resection (upper boundary, 90.7 *versus* 76.7%, respectively; lower boundary, 32 *versus* 25%, respectively). In individual primary studies, there was also a tendency towards improved short-term mortality in the BTS cohorts, which was also reported in four of the identified systematic reviews^19,20,47,48^. There are inherent biases when interpreting these results. First, there were no identified randomized trials. That being said, the largest primary study in this review^25^ used propensity score matching and confirmed that a BTS approach using SEMS statistically reduced morbidity and stoma formation rates, while also increasing rates of laparoscopic surgery. Mortality was also lower for the SEMS group in that study^25^, but was just outside of statistical significance (*P* = 0.069). Second, the vast majority of studies reporting BTS in this review had reduced sample sizes. Finally, the worse outcomes reported in patients undergoing emergency resection may be due to the physiological status of the patient necessitating emergency intervention, rather than awaiting SEMS placement. The favourable stent outcomes could also be explained by the selective identification of patients, with those patients deemed ‘difficult’ to stent managed surgically.

The vast majority of BTS patients within this review underwent SEMS as a BTS rather than a defunctioning stoma. SEMS is a relatively new endoscopic intervention and thus its role is not fully established, particularly regarding the feasibility of its use in proximal cancers. There are organizational-level difficulties in access to emergency endoscopy services, as well as educational deficits in endoscopy training, which may limit access to clinicians trained in proximal SEMS placement^62–64^. Although a defunctioning stoma is an invasive surgical procedure, it carries fewer risks than major colon resection and can often be performed laparoscopically. The creation of a defunctioning stoma to allow for accurate staging and the potential use of neoadjuvant chemotherapy is well established for distal colon and rectal cancers^50,62,65–67^. However, there is limited evidence to support the use of a defunctioning stoma in ROCCs, which reflects why surgeons often opt to perform an emergency resection in this setting. Although a limited number of patients underwent the creation of a defunctioning stoma in this review, the findings demonstrate how a stoma as a BTS can lead to a longer time interval until definitive resection compared with SEMS. This may provide an ideal opportunistic window for the future use of neoadjuvant chemotherapy. It is therefore essential that, to optimize long-term patient oncological outcomes, future studies investigate the optimal surgical approach when managing patients with ROCCs. Studies should investigate the outcomes associated with emergency resection *versus* BTS, as well as differing BTS approaches (SEMS *versus* stoma), including the feasibility for enabling neoadjuvant chemotherapy.

There are limitations to this study. First, the literature was restricted to studies published during and after the year 2000, which may have excluded a number of studies that would otherwise have met the inclusion criteria. This decision was made because SEMS is a relatively new technique and it was expected that including studies published before 2000 may bias results given the initial learning curve of the intervention and because surgical techniques have progressed, in particular the use of laparoscopic surgery in emergency patients, and the aim of this review was to encapsulate data that would inform modern-day practice. Second, there was no formal aggregation or meta-analysis of outcomes. This is beyond the remit of a scoping review, and the aim of the study was to report strengths and deficits in the current literature. A formal assessment of study quality was not conducted because this is outside the remit of a scoping review^23,24^. Finally, the strength of the conclusions is supported by poorly powered primary research studies.

In summary this study provides a detailed overview of the current body of literature in managing ROCCs. Included studies lack methodological quality and BTS approaches focus on SEMS, rather than established surgical alternatives (defunctioning stoma). The findings of the present study do imply that a BTS approach reduces perioperative morbidity, which theoretically should increase the adoption of adjuvant chemotherapy, but the included studies are not powered adequately to definitively support this conclusion. In addition, with recent oncological trial data suggesting the benefits of neoadjuvant chemotherapy, a BTS approach may permit neoadjuvant chemotherapy for future patients presenting acutely, which is not possible with emergency surgery. Long-term oncological outcomes are not rigorously reported, which will form a key aspect in assessing the potential utility of a BTS approach in practice.

Overall, a BTS approach seems to confer preferable perioperative outcomes compared with emergency surgery. The BTS literature is poorly powered and limited by selection bias, meaning the current evidence is insufficient to conclude true superiority to the BTS approach. It is recommended that further high-quality research is undertaken to ascertain the feasibility of a BTS approach in clinical practice, including acceptability to patients. This will inform the design and delivery of a definitive trial.

## Supplementary Material

zraf129_Supplementary_Data

## Data Availability

All relevant data are presented in the review with no further data to share.
