# Supplementary material for: Management of right-sided obstructing colon cancers: scoping review
Source: BJS Open. 2025 Nov 4;9(6):zraf129. doi: 10.1093/bjsopen/zraf129 (PMC12587153; doi:10.1093/bjsopen/zraf129)
Supplement: zraf129_Supplementary_Data [file zraf129_supplementary_data.docx]

**Management of right-sided obstructing colonic cancers: a scoping review**

Baker DM^1^, Aimar K^2^, Jacobs S^2^, Lee MJ^3^

Affiliations:

1. Leeds Institute of Emergency General Surgery, University Hospitals Leeds, UK
2. Department of Trauma and Emergency General Surgery, University Hospitals of Birmingham, UK
3. Department of Applied Health Sciences, College of Medicine and Health, University of Birmingham

**Corresponding author.** Mr Matthew Lee, m.j.lee.1@bham.ac.uk

**Supplementary Materials - Index**

| **Supplementary Figures and Tables** |  |
| --- | --- |
| Detailed search strategies table S1-S3 | *page 2-4* |
| Supplementary table S4 – Complete list of patient descriptors | *page 5-11* |

**Supplementary Figures and Tables**

Supplementary table S1 – Ovid Medline search

| **Database:** | **Ovid MEDLINE(R) ALL <1946 to January 13, 2025>** | **Results per line:** |
| --- | --- | --- |
| **Date:** | **14/01/2025** |  |
| 1 | Colonic Diseases/ | 16627 |
| 2 | exp colonic neoplasms/ | 85356 |
| 3 | ((cancer* or neoplasm* or tumo?r* or metastas?s) adj2 (colon* or splenic* or sigmoid* or intestine*)).ti,ab,kw,kf. | 87936 |
| 4 | 1 or 2 or 3 | 146945 |
| 5 | (obstruction* adj4 (colon* or splenic* or sigmoid* or intestine*)).ti,ab,kw,kf. | 4636 |
| 6 | Intestinal Obstruction/ | 32134 |
| 7 | 5 or 6 | 34301 |
| 8 | 4 and 7 | 4591 |
| 9 | (right or "right-side*" or "right side*" or rightside*).ti,ab,kw,kf. | 668648 |
| 10 | 8 and 9 | 355 |
| 11 | Colectomy/ | 20908 |
| 12 | Ileostomy/ | 7518 |
| 13 | (resection* or surger* or surgical* or operat* or colectom* or ileostom* or procedure*).ti,ab,kw,kf. | 4397516 |
| 14 | 11 or 12 or 13 | 4403146 |
| 15 | Stents/ | 81166 |
| 16 | Self Expandable Metallic Stents/ | 1428 |
| 17 | Surgical Stomas/ | 2664 |
| 18 | (stent* or SEMS or "bridge* to surger*" or BTA or stoma).ti,ab,kw,kf. | 148455 |
| 19 | 15 or 16 or 17 or 18 | 163788 |
| 20 | 14 or 19 | 4489354 |
| 21 | 10 and 20 | 291 |
| 22 | Emergencies/ | 44113 |
| 23 | Acute Care Surgery/ | 148 |
| 24 | Emergency Service, Hospital/ | 93274 |
| 25 | (acute or emergenc*).ti,ab,kw,kf. | 2018661 |
| 26 | 22 or 23 or 24 or 25 | 2047374 |
| 27 | 10 and 20 and 26 | 135 |
| 28 | limit 27 to yr="2000 - 2025" | 82 |
| 29 | limit 28 to english language | 71 |

Supplementary table S2 – Embase search

| **Database:** | **Embase <1974 to 2025 Week 02>** | **Results per line:** |
| --- | --- | --- |
| **Date:** | **14/01/2024** |  |
| 1 | exp colon tumor/ | 182819 |
| 2 | colon disease/ | 10055 |
| 3 | ((cancer* or neoplasm* or tumo?r* or metastas?s) adj2 (colon* or splenic* or sigmoid* or intestine*)).ti,ab,kw,kf. | 122086 |
| 4 | 1 or 2 or 3 | 233303 |
| 5 | intestine obstruction/ | 37266 |
| 6 | (obstruct* adj4 (colon* or splenic* or sigmoid* or intestine*)).ti,ab,kw,kf. | 7087 |
| 7 | 5 or 6 | 42477 |
| 8 | 4 and 7 | 4977 |
| 9 | (right or "right-side*" or "right side*" or rightside*).ti,ab,kw,kf. | 989638 |
| 10 | 8 and 9 | 715 |
| 11 | exp colectomy/ | 63776 |
| 12 | ileostomy/ | 18643 |
| 13 | exp colon/ and surgery/ | 10896 |
| 14 | (resection* or surger* or surgical* or operat* or colectom* or ileostom* or procedure*).ti,ab,kw,kf. | 5686079 |
| 15 | 11 or 12 or 13 or 14 | 5703816 |
| 16 | self expanding stent/ | 5053 |
| 17 | stent/ | 99869 |
| 18 | stoma/ | 15099 |
| 19 | colon stoma/ | 226 |
| 20 | (stent* or SEMS or "bridge* to surger*" or BTA or stoma).ti,ab,kw,kf. | 245864 |
| 21 | 16 or 17 or 18 or 19 or 20 | 264554 |
| 22 | 15 or 21 | 5828743 |
| 23 | 10 and 22 | 653 |
| 24 | Emergency/ | 55339 |
| 25 | oncologic emergency/ | 62 |
| 26 | emergency care/ | 65835 |
| 27 | Emergency treatment/ | 18474 |
| 28 | (acute or emergenc*).ti,ab,kw,kf. | 2783565 |
| 29 | 24 or 25 or 26 or 27 or 28 | 2811937 |
| 30 | 10 and 22 and 29 | 258 |
| 31 | limit 30 to yr="2000 - 2025" | 231 |
| 32 | limit 31 to english language | 216 |

Supplementary table S3 – CENTRAL and CDSR search

| **Database:** | **Cochrane Central Register of Controlled Trials (CENTRAL) and Cochrane Database of Systematic Reviews (CDSR)** | **Results per line:** |
| --- | --- | --- |
| **Date:** | **14/01/2024** |  |
| #1 | MeSH descriptor: [Colonic Diseases] this term only | 448 |
| #2 | MeSH descriptor: [Colonic Neoplasms] explode all trees | 2612 |
| #3 | ((cancer* or neoplasm* or tumo?r* or metastas?s) NEAR/2 (colon* or splenic* or sigmoid* or intestine*)):ti,ab,kw | 7587 |
| #4 | #1 or #2 or #3 | 7989 |
| #5 | MeSH descriptor: [Intestinal Obstruction] this term only | 457 |
| #6 | ((obstruction*) NEAR/4 (colon* or splenic* or sigmoid* or intestine*)):ti,ab,kw | 1037 |
| #7 | #5 or #6 | 1378 |
| #8 | #4 and #7 | 193 |
| #9 | (right or (right-side*) or (right NEXT side*) or rightside*):ti,ab,kw | 39878 |
| #10 | #8 and #9 | 9 |
| #11 | MeSH descriptor: [Colectomy] this term only | 932 |
| #12 | MeSH descriptor: [Ileostomy] this term only | 316 |
| #13 | (resection* or surger* or surgical* or operat* or colectom* or ileostom* or procedure*):ti,ab,kw | 626506 |
| #14 | #11 or #12 or #13 | 626506 |
| #15 | MeSH descriptor: [Stents] this term only | 4699 |
| #16 | MeSH descriptor: [Self Expandable Metallic Stents] this term only | 87 |
| #17 | MeSH descriptor: [Surgical Stomas] this term only | 165 |
| #18 | (stent* or SEMS or (bridge* to surger*) or BTA or stoma):ti,ab,kw | 22363 |
| #19 | #16 or #17 or #18 | 22381 |
| #20 | #14 or #19 | 635660 |
| #21 | #10 and #20 | 9 |
| #22 | MeSH descriptor: [Emergencies] this term only | 1858 |
| #23 | MeSH descriptor: [Acute Care Surgery] this term only | 1 |
| #24 | MeSH descriptor: [Emergency Service, Hospital] this term only | 3805 |
| #25 | (acute or emergenc*):ti,ab,kw | 215493 |
| #26 | #22 or #23 or #24 or #25 | 215493 |
| #27 | #21 and #26 | 1 |

Supplementary table S4 – Complete list of patient descriptors with number of times reported and citing study.

| Descriptor | Number of studies reporting | Study |
| --- | --- | --- |
| *Patient descriptors* |  |  |
| Age | 21 | Amelung 2016 Boeding 2023 Faucheron 2018 Hotta 2012 Huang 2024 Ji 2017 Kye 2016 Li 2020 Li 2015 Lockhorst 2025 Mege 2018 Mege 2019 Morita 2019 Ng 2008 Rosander 2021 Sakamoto 2020 Suzuki 2013 van den Berg 2014 Yao 2011 Zeng 2021 Tan 2010 |
| Gender | 21 | Amelung 2016 Boeding 2023 Faucheron 2018 Hotta 2012 Huang 2024 Ji 2017 Kye 2016 Li 2020 Li 2015 Lockhorst 2025 Mege 2018 Mege 2019 Morita 2019 Ng 2008 Rosander 2021 Sakamoto 2020 Suzuki 2013 van den Berg 2014 Yao 2011 Zeng 2021 Tan 2010 |
| ASA | 14 | Amelung 2016 Boeding 2023  Faucheron 2018 Huang 2024 Ji 2017 Kye 2016 Lockhorst 2025 Mege 2018 Mege 2019 Morita 2019 Ng 2008 Rosander 2021 Zeng 2021 Tan 2010 |
| Comorbidity | 8 | Huang 2024 Li 2020 Lockhorst 2025 Mege 2018 Mege 2019 Ng 2008 Sakamoto 2020 Tan 2010 |
| Charlson Comorbidity Index | 3 | Rosander 2021 Sakamoto 2020 van den Berg 2014 |
| POSSUM score | 1 | Hotta 2012 |
| SNAQ score | 1 | Lockhorst 2025 |
| ECOG performance status | 1 | Mege 2019 |
| BMI | 9 | Huang 2024 Ji 2017 Li 2015 Lockhorst 2025 Mege 2018 Ng 2008 Sakamoto 2020 Suzuki 2013 Zeng 2021 |
| Smoking status | 2 | Lockhorst 2025 Sakamoto 2020 |
| Operation history (previous abdominal surgery) | 2 | Li 2015 Ng 2008 Zeng 2021 |
| Obstruction duration | 2 | Li 2015 Ng 2008 |
| Time from obstruction to admission | 1 | Huang 2024 |
| Time from presentation to intervention | 1 | Huang 2024 |
| Degree of obstruction (partial / complete) | 2 | Huang 2024 Morita 2019 |
| Haemodynamic instability at admission | 3 | Mege 2018 Mege 2019 Genser 2020 |
| Hb | 3 | Faucheron 2018 Lockhorst 2025 Tan 2010 |
| WCC | 2 | Faucheron 2018 Tan 2010 |
| CRP | 1 | Lockhorst 2025 |
| CEA | 3 | Hotta 2012 Kye 2016 Lockhorst 2025 |
| Urea | 1 | Tan 2010 |
| Creatinine | 1 | Tan 2010 |
| Pre-op nutriton (TPN / probe feeding) | 2 | Boeding 2023 Lockhorst 2025 |
| Pre-operative metastases | 1 | Boeding 2023 |
| CT scan (whether this was performed) | 1 | Tan 2010 |
| CT features (absence of bowel wall enhancement, pneumoperitoneum) | 1 | Genser 2020 |
| *Intervention/technicality descriptors* |  |  |
| Urgency of surgery | 3 | Amelung 2016 Boeding 2023 Lockhorst 2025 |
| Surgical approach (open / laparoscopic / conversion to laparotomy) | 14 | Amelung 2016 Boeding 2023 Lockhorst 2025 van den Berg 2014 Huang 2024 Ji 2017 Kye 2016 Li 2020 Li 2015 Morita 2019 Ng 2008 Sakamoto 2020 Suzuki 2013 Zeng 2021 |
| Surgical procedure performed | 10 | Amelung 2016 Boeding 2023 Lockhorst 2025 van den Berg 2014 Genser 2020 Tan 2010 Huang 2024 Morita 2019 Ng 2008 Yao 2011 |
| Length of incision | 2 | Li 2015 Ng 2008 |
| Intra-op enteral decompression | 1 | Li 2015 |
| Duration of operation | 9 | Huang 2024 Ji 2017 Kye 2016 Li 2020 Li 2015 Morita 2019 Ng 2008 Suzuki 2013 Zeng 2021 |
| Blood loss | 9 | Huang 2024 Ji 2017 Kye 2016 Li 2020 Li 2015 Morita 2019 Ng 2008 Suzuki 2013 Zeng 2021 |
| Combined resection performed | 1 | Kye 2016 |
| Surgical strategy (one-stage vs two-stage) | 1 | Faucheron 2018 |
| Primary anastomosis constructed | 3 | Amelung 2016 Huang 2024 Zeng 2021 |
| Stoma formation | 9 | Amelung 2016 Rosander 2021 Tan 2010 Huang 2024 Ji 2017 Li 2020 Morita 2019 Sakamoto 2020 Zeng 2021 |
| Interval between stent/stoma and resection | 4 | Amelung 2016 Huang 2024 Sakamoto 2020 Zeng 2021 |
| Perforated primary tumour | 4 | Mege 2018 Genser 2020 Li 2020 Mege 2018 |
| Unresectable primary tumour | 1 | Mege 2018 |
| Intra-operative complication | 2 | Ji 2017 Kye 2016 |
| Temporary discharge after stenting | 1 | Sakamoto 2020 |
| Surgeon experience | 2 | Rosander 2021 Ng 2008 |
| Hospital factors (low vs high volume, unversity hospital vs other) | 2 | Rosander 2021 Sakamoto 2020 |
| Observation period | 1 | Suzuki 2013 |
| Colonic ischaemia | 1 | Mege 2018 |
| Caecal perforation | 1 | Mege 2018 |
| Status of caecum | 1 | Tan 2010 |
| Associated obstructive colitis | 1 | Morita 2019 |
| Ileocaecal valve competency | 1 | Tan 2010 |
| Ascites | 1 | Li 2020 |
| Resection of caecum (yes/no) | 1 | Tan 2010 |
| Peritonitis | 2 | Mege 2018 Genser 2020 |
| *Surgical oncology & histopathological descriptors* |  |  |
| Tumour location | 11 | Amelung 2016 Boeding 2023 Huang 2024 Ji 2017 Li 2020 Morita 2019 Sakamoto 2020 Suzuki 2013 Yao 2011 Zeng 2021 Tan 2010 |
| Staging | 14 | Hotta 2012 Huang 2024 Ji 2017 Li 2020 Li 2015 Morita 2019 Ng 2008 Sakamoto 2020 Suzuki 2013 van den Berg 2014 Tan 2010 Faucheron 2018 Mege 2018 Zeng 2021 |
| Synchronous metastases | 4 | Boeding 2023 Mege 2018 Mege 2019 Lockhorst 2025 |
| Metachronous metastasis | 1 | Lockhorst 2025 |
| Location of metachronous metastasis | 1 | Lockhorst 2025 |
| Histopathological type | 4 | Hotta 2012 Li 2020 Suzuki 2013 Huang 2024 |
| Tumour differentiation | 2 | van den Berg 2014 Kye 2016 |
| Tumour size | 4 | Hotta 2012 Li 2020 Li 2015 Ng 2008 |
| pT stage | 4 | Amelung 2016 Hotta 2012 Lockhorst 2025 Kye 2016 |
| pN stage | 4 | Amelung 2016 Lockhorst 2025 Rosander 2021 Kye 2016 |
| M1 stage | 2 | Amelung 2016 Lockhorst 2025 |
| cT4 stage | 1 | Mege 2018 |
| Length of specimen | 1 | Faucheron 2018 |
| Number of harvested lymph nodes | 12 | Ng 2008 Rosander 2021 van den Berg 2014 Faucheron 2018 Huang 2024 Ji 2017 Kye 2016 Li 2020 Li 2015 Mege 2018 Suzuki 2013 Zeng 2021 |
| Number of positive lymph nodes harvested | 3 | Huang 2024 Kye 2016 Li 2020 |
| Resection (radical vs non-radical) | 1 | Rosander 2021 |
| Proximal resection margin | 1 | Kye 2016 |
| Distal resection margin | 1 | Kye 2016 |
| Resection (R0 / R1 / R2) | 5 | van den Berg 2014 Amelung 2016 Faucheron 2018 Li 2020 Morita 2019 |
| Lymphatic invasion | 6 | Hotta 2012 Li 2020 Suzuki 2013 Faucheron 2018 Kye 2016 Mege 2018 |
| Vascular invasion | 6 | Hotta 2012 Li 2020 Suzuki 2013 Faucheron 2018 Huang 2024 Kye 2016 |
| Perineual invasion | 4 | Faucheron 2018 Huang 2024 Kye 2016 Mege 2018 |
| Vascular emboli | 1 | Faucheron 2018 |
